# Use of a plant-based polysaccharide hemostat for the treatment of sternal bleeding after median sternotomy

**DOI:** 10.1186/s13019-015-0263-4

**Published:** 2015-04-24

**Authors:** Christoph Schmitz, Ralf Sodian

**Affiliations:** Department of Cardiac Surgery, University of Munich, 81377 Munich, Germany

**Keywords:** Bleeding control, Sternotomy, Polysaccharide hemostats

## Abstract

**Background:**

In cardiac surgery profuse or persistent sternal bleeding after sternotomy is routinely controlled with bone wax. However, bone wax should be avoided, especially in high-risk patients for nonunion of the sternum and infections. Purpose of this study was to evaluate an alternative technique to control bleeding after medium sternotomy using a plant based absorbable polysaccharide hemostat.

**Methods:**

A consecutive series of 38 patients requiring median sternotomy for coronary artery bypass surgery (21 OPCAB, 17 CABG) had sternal bleeding control with the polysaccharide hemostat, STARSIL® HEMOSTAT. This hemostat is a hydrophilic powder, which achieves hemostasis after topical application at the surgical wound site. Initially it dehydrates blood rapidly, thus accelerating aggregation of platelets and blood solids. Thereafter, it forms a gelled adhesive matrix, which serves as a mechanical barrier against further bleeding. The polysaccharide is completely resorbed within 48 to 72 hours.

**Results:**

Satisfactory control of sternal bleeding was observed in 37 patients (97%). No product-related complications were observed or any other major adverse events in an observation period of 3 months.

**Conclusion:**

Polysaccharide hemostats appear to be safe and effective for bleeding control of the sternum.

## Background

Profuse bleeding or persistent oozing after sternotomy is routinely controlled with bone wax. However, bone wax, which cannot be absorbed by the body, has been shown to inhibit osseous fusion, promote infections, and elicit inflammatory reactions [1-3]. Nowadays, an increasing number of patients requiring sternotomy are at high risk for sternal instability and wound healing complications. Therefore improved methods for bleeding control of the sternum without using bone wax are demanded [4,5]. We present here an alternative to control sternal bleeding after sternotomy using a hemostatic powder.

## Methods

A consecutive series of 38 patients requiring median sternotomy for coronary artery surgery (21 OPCAB and 17 CABG) were included into the study. 35 patients presented with preoperative platelet inhibition (21 single and 14 dual platelet inhibition). Additional demographic patent data are listed in Table 1. For study purposes surgeons rated the performance of STARSIL® HEMOSTAT using a visual analogue scale (VAS) from 1 to 10. One was a very bad, 10 a perfect performance of the product. All patients were followed-up for three months.

**Table 1:** **Demographic data of patients receiving STARSIL® HEMOSTAT for sternal bleeding control**

OPCAB (n)	21
CABG (n)	17
Age (years)	67 ± 14
RITA/LITA (n)	15
COPD (n)	14
Diabetes (n)	16
Severe osteoporosis (n)	9
BMI > 30 (n)	6
Platelet inhibition	
No	3
Single (ASA)	21
Dual	14

OPCAB: off-pump coronary artery bypass; CABG: coronary arterial bypass grafting; RITA: right internal thoracic artery; LITA: left internal thoracic artery; COPD: chronic obstructive pulmonary disease; BMI: body mass index; ASA: acetyl salicylic acid.

### Study product

STARSIL® HEMOSTAT (HEMOTEC MEDICAL GmbH, Velen, Germany) is a hemostat consisting of 5 g purified plant-based absorbable polysaccharide that can be administered to the entire operation area. It is a “second generation” starch-based hemostat. When compared to a “first generation” product, it has distinct advantages, e.g. significantly increased water absorption quantity (here: 64 ml/2 g powder). The powder is available off-the-shelf without any further preparation. In order to obtain hemostasis it can be applied directly onto a bleeding wound. The hemostatic effect results from rapid dehydration and subsequent concentration of blood components like red blood cells, platelets and serum proteins (thrombin, fibrinogen, etc.), thus accelerating the clotting cascade. As a result a gelled adhesive matrix is produced. Normal platelet activation and fibrin deposition produce a clot that functions as a mechanical barrier and limits further bleeding. Absorption of the particles is achieved within approximately 48 to 72 hours. STARSIL® HEMOSTAT is biocompatible, non-pyrogenic and does not contain any allo- or xenogenic additions.

### Application technique

**Figure 1:**
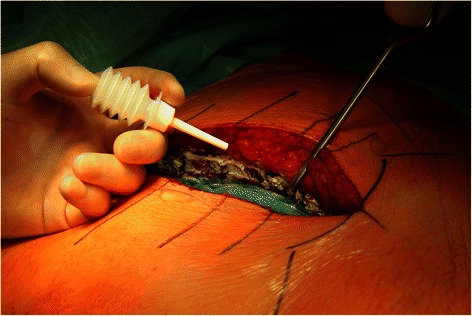
STARSIL® HEMOSTAT applied on both sides of the sternum after median sternotomy.

**Figure 2:**
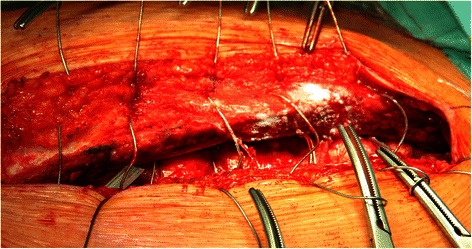
STARSIL® HEMOSTAT applied on both sides of the sternum after sternotomy before closure with steel wires.

Skin incision and median sternotomy were performed with standard techniques trying to use electrocautery sparingly. Directly after sternotomy STARSIL® HEMOSTAT was applied on each side of the sternal spongiosa (Figure 1). Towels were wrapped around the sternum for atraumatic tissue treatment before insertion of the retractor. Bleeding control was rated satisfactory, when bleeding from the sternal spongiosa was achieved within one minute. After sternotomy patients were fully heparinized (activated clotting time > 400 seconds) and open-heart surgery was performed in a routine fashion. After graft placement protamine was administered in order to reverse the heparin effect. Before sternal closure residual STARSIL® HEMOSTAT was applied on both sides of the sternum (Figure 2). Sternum was closed with sternal wires, subcutaneous fat and skin with absorbable sutures.

## Results

Satisfactory control of sternal bleeding was observed in 37 cases (97%). In all but two patients a single application of the hemostat led to satisfactory results. The two additional patients showed persistent bleeding requiring a second application of STARSIL® HEMOSTAT. One surgeon rated a second application as “non satisfactory” while the other had no problem using the powder a second time. Surgeon’s general satisfaction according to the VAS was 8.4 ± 1.4 (Table 2).

**Table 2:** **Intra- and postoperative results of patients receiving STARSIL® HEMOSTAT for sternal bleeding control**

Satisfactory bleeding control (n)	37 (97%)
Hemoglobin	
Preop. (g/dL)	12.6 ± 1.2
Direct postop. (g/dL)	9.1 ± 1.8
On discharge (g/dL)	12.7 ± 1.6
Drainage 6 h (mL)	480 ± 380
Cell saver (ml)	360 ± 210
Blood units (U)	26/19 pts
Result of VAS	8.4 ± 1.4

VAS: visual analogue scale.

No adverse events or allergic reactions were observed during the study period. There were no cases of in-hospital mortality or product related morbidity observed during the 3-month follow-up. No re-operations due to graft alteration, bleeding or unstable sternum were necessary. No patient required sternal re-fixation due to instability. Two patients experienced superficial wound infections. The first patient required an outpatient procedure in which the wound was cleaned. Subsequently, secondary wound-closure was performed. In the second patient the wound was treated conservatively with daily wound dressings for three weeks. Another patient was re-administered six weeks postoperatively for sternal wire extraction due to chest pain. All postoperative events were rated as not product related.

## Discussion

Surgeons commonly recommend to avoid the use of bone wax for control of sternal bleeding, especially in patients at a high risk for infection or nonunion [6]. Furthermore, bone wax is often ineffective in elderly patients and those with osteoporosis; additionally, the spongiosa scaffold of the sternum is destroyed when applying bone wax, which may result in the marrow cavity absorbing large quantities of wax without hemostasis. Despite the negative effects associated with the use of bone wax, the product remains in widespread use, presumably due to the perceived lack of suitable alternatives. In our study STARSIL® HEMOSAT proved to be a simple, safe and effective alternative for bleeding control in patients undergoing median sternotomy. Results were very satisfactory in most cases, even when applied under difficult conditions, e.g. when dual platelet inhibition was necessary.

Other hemostatic agents like fibrin sealant or microfibrillar collagen may also be helpful. But when compared to polysaccharide hemostats they are relatively expensive. Furthermore, it has been published that the use of fibrin glue may increase morbidity in cardiac surgery [7]. Microfibrillar collagen has also been used to reduce sternal bleeding. However, due to the small diameter of its needle-shaped structure, there is a risk for passing filters of blood salvage system, thus entering patient’s circulation, and possibly resulting in organ damage [8].

Starch-based hemostats are in clinical use for more than 10 years. STARSIL® HEMOSTAT is a “second generation” starch-based hemostat. When compared to Arista (Medafor Inc., Minneapolis, MN, USA), a “first generation” product, STARSIL® HEMOSTAT has distinct advantages, e.g. significantly increased water absorption quantity.

Another interesting feature of starch-based hemostats seems to be an adhesion barrier function, which was described in an animal model [9]. In a study on Wister outbred rats a relationship between inflammation and tissue necrosis as well as the possible formation of adhesions could be demonstrated. Six different hemostatic agents were tested. The starch-based hemostat proved to produce significantly lower adhesion formation when compared to control (p < 0.05).

### Limitations

There are some limitations that should be pointed out: In our observation we had two different patient groups with either on- or off-pump surgical revascularization. Therefore the study represents small patient groups with a relatively short median follow-up time. The study reflects as well a single center experience of two experienced surgeons. We already planned a larger multicenter trial with more patients which will confirm the clinical safety and efficacy of STARSIL® HEMOSTAT in all surgical settings. There are some polysaccharide based hemostatic powders on the market that already demonstrated advantages and limitations when compared to other hemostatic agents. Therefore we did not compare different groups in this study. Furthermore the primary endpoint was safety of use of STARSIL® HEMOSTAT when applied for bleeding control of the sternum.

## Conclusion

In conclusion, polysaccharide hemostats, like STARSIL® HEMOSTAT, appear to be safe and effective controlling sternal bleeding in cardiac surgery. Furthermore, they may help to reduce wound-healing complications associated with use of bone wax.

## Consent

Written informed consent was obtained from the patient for the publication of this report and any accompanying images.

## Abbreviation

VAS: Visual analogue scale
